# Commentary: Enhanced Monitoring of the Preterm Infant during Stabilization in the Delivery Room

**DOI:** 10.3389/fped.2016.00064

**Published:** 2016-06-14

**Authors:** David J. R. Hutchon

**Affiliations:** ^1^Darlington Memorial Hospital, Darlington, UK

**Keywords:** neonatal heart rate, first minute after birth, Doppler ultrasound, auscultation, caesarean section

Finn et al. present an excellent summary of current and emerging methods by which to monitor preterm infants during stabilization in the delivery room, but I disagree with their characterization of the usefulness of Doppler ultrasound, and they fail to comment on the impact of delayed cord clamping on heart rate in the first minutes of postnatal life. Furthermore, I believe recent work using Electrical Cardiometry ICON device (1) to measure HR may shed light on the impact of the timing of cord clamping on postnatal HR, a topic not sufficiently discussed in this review. The need for improved monitoring documentation and assessment is not confined to the preterm, and, although less critically, applies also to the term neonate. Although there is a clear need for such monitoring in the preterm infant, the same standards have to be available for every neonate in whom assistance during stabilization is being considered. The authors point out that the heart rate is the only truly objective parameter that can be measured and documented in the first minute or so. The pros and cons of oximetry and ECG are presented but determining the heart rate in the first minute when intervention is considered most critical is not possible by either of these technologies.

Doppler heart rate monitoring is a well-established standard for the fetal heart rate. The authors state that with Doppler ultrasound “*HR assessments in the DR are accurate compared with clinical and pulse oximetry assessments* (2).” The Phillipos paper, which they reference, simply states that “*Doppler ultrasound shows potential for clinical use, however future evidence is needed to support this conclusion*.” Phillipos et al. (2) do in fact reference some original evidence showing that a reliable signal can be obtained immediately after placing the transducer on the newborn’s precordium and within the first minute after birth (3, 4). At the Fifth Annual Meeting of Cork Neonatal Research Group (5) in Ireland during 2013, I demonstrated that Doppler ultrasound was able to provide heart sounds, a calculated heart rate, and function from the moment of application. I showed that it will also function through the polyethylene wrap for a preterm neonate. Both ECG and oximetry require exposure of the neonate’s skin for the probes to function thus reducing the efficacy of the polythene wrap. A small Doppler fetal monitor can also be used at cesarean section when the Doppler equipment is placed inside a sterile polyethylene bag. Either the heart rate can be read and documented directly from the Doppler display or can be connected *via* Bluetooth to an external recorder. This allows proper assessment and documentation of the neonatal heart rate at cesarean section and facilitates delayed cord clamping and resuscitation with the cord intact. Doppler ultrasound is established as a method for measuring and documenting the fetal heart rate, so it is hard to argue that this will not apply moments later when the fetus is ex-utero as a neonate.^1^

Finn et al. state “However, clinical experience is required for accurate assessments and continuous measurements are not practical.” After the first minute or so when oximetry is functioning, precordial Doppler is redundant but continuous measurements using a lightweight transducer makes continued hands-free measurements perfectly practical for several minutes after birth.

What is the normal heart rate for term and preterm neonate during the first minutes after birth? Finn et al. point out that the standard charts (6) were derived from a population of neonates in whom transition was interrupted by early cord clamping. Early cord clamping was a standard obstetrical practice at that time. Although Finn et al. do not specifically discuss the standard of care of the neonate, it goes without saying that they would expect the care to be optimal. Increasing evidence shows that optimal care of the neonate will involve keeping the umbilical cord and the placental circulation intact during transition. A study of the neonatal heart rate has been repeated with the standard midwifery practice of delayed cord clamping and placing the neonate on the mother’s abdomen “skin to skin” during this time. The new study showed higher oxygen saturation in the neonates with delayed cord clamping but no difference in the heart rates (7). Katheria et al. explored these criteria in the NICOM study by placing the neonate on a specially designed mobile resuscitation trolley, which allowed the neonate to lie on a smooth warm surface (equivalent to skin to skin) and with the facility to initiate ventilation if the need arose (8). They found that heart rate did not significantly change over 5 min, and the mean rate at 1 min was 176 bpm (SD 15.3). This is 15 bpm higher than the accepted normal fetal heart range but very mush less disparate from the normal range than the Dawson/Smit standards. Dawson showed that 10% of their babies will have a heart rate below 50 bpm, below the 60 bpm threshold for providing external cardiac massage! None of the babies in the Katheria series had a heart rate under 110 bpm. Is this anomaly explained by the small numbers in the Katheria study (20 babies) in comparison to the Dawson (308 babies) and Smit (109 babies) studies? We await further and larger studies using techniques, such as Doppler ultrasound and NICOM, which will determine the neonatal heart rate from the moment of birth.

## Author Contributions

The author confirms being the sole contributor of this work and approved it for publication.

## Conflict of Interest Statement

The author declares that the research was conducted in the absence of any commercial or financial relationships that could be construed as a potential conflict of interest.

## Footnotes

^1^https://www.youtube.com/watch?v=ut0vpZxkidI
